# O-GlcNAc transferase promotes glioblastoma by modulating genes responsible for cell survival, invasion, and inflammation

**DOI:** 10.1016/j.jbc.2023.105235

**Published:** 2023-09-09

**Authors:** Muhammad Abid Sheikh, Thilina T. Alawathugoda, Garima Vyas, Bright Starling Emerald, Suraiya A. Ansari

**Affiliations:** 1 Department of Biochemistry and Molecular Biology, College of Medicine and Health Sciences, United Arab Emirates University, Al Ain, Abu Dhabi, United Arab Emirates; 2 Department of Anatomy, College of Medicine and Health Sciences, United Arab Emirates University, Al Ain, Abu Dhabi, United Arab Emirates; 3 Zayed Center for Health Sciences, United Arab Emirates University, Al Ain, Abu Dhabi, United Arab Emirates; 4 Precision Medicine Research Institute Abu Dhabi (PMRIAD), United Arab Emirates University, Al Ain, Abu Dhabi, United Arab Emirates

**Keywords:** O-GlcNAcylation, gene expression, glioblastoma, transcription regulation, metabolism

## Abstract

Metabolic reprogramming has emerged as one of the key hallmarks of cancer cells. Various metabolic pathways are dysregulated in cancers, including the hexosamine biosynthesis pathway. Protein O-GlcNAcylation is catalyzed by the enzyme O-GlcNAc transferase (OGT), an effector of hexosamine biosynthesis pathway that is found to be upregulated in most cancers. Posttranslational O-GlcNAcylation of various signaling and transcriptional regulators could promote cancer cell maintenance and progression by regulating gene expression, as gene-specific transcription factors and chromatin regulators are among the most highly O-GlcNAcylated proteins. Here, we investigated the role of OGT in glioblastoma. We demonstrate that OGT knockdown and chemical inhibition led to reduced glioblastoma cell proliferation and downregulation of many genes known to play key roles in glioblastoma cell proliferation, migration, and invasion. We show that genes downregulated due to OGT reduction are also known to be transcriptionally regulated by transcriptional initiation/elongation cofactor BRD4. We found BRD4 to be O-GlcNAcylated in glioblastoma cells; however, OGT knockdown/inhibition neither changed its expression nor its chromatin association on promoters. Intriguingly, we observed OGT knockdown led to reduced Pol II-Ser2P chromatin association on target genes without affecting other transcription initiation/elongation factors. Finally, we found that chemical inhibition of BRD4 potentiated the effects of OGT inhibition in reducing glioblastoma cell proliferation, invasion, and migration. We propose BRD4 and OGT act independently in the transcriptional regulation of a common set of genes and that combined inhibition of OGT and BRD4 could be utilized therapeutically for more efficient glioblastoma cell targeting than targeting of either protein alone.

Metabolic reprogramming has emerged as a key hallmark of cancer cell progression and metastasis (1, 2). “Warburg effect” was observed in cancer cells first by Otto Warburg who recognized the reliance of cancer cells on glycolysis rather than oxidative phosphorylation for energy (3). However, the role of aerobic glycolysis as it has been termed in the regulation of cancer remained unclear until recently. It has been proposed that cancer cells and probably all types of proliferating cells rely on increased nutrient uptake and its utilization for biomass production to meet the demand for active cell proliferation (3). Indeed, several signaling pathways altered in cancer facilitate increased nutrient uptake and macromolecular biosynthesis (4). These pathways such as PI3K/AKT/mTOR induce metabolite flux toward aerobic glycolysis, lipid and amino acid biosynthesis, and glutamine metabolism (4), thus promoting tumor cell maintenance and adaptation to microenvironment. Such extensive metabolic changes in cancer cells are also associated with extensive transcriptomic and proteomic alterations to meet the requirements of metabolic reprogramming (5, 6). However, how do metabolic alterations in cancer affect gene expression and *vice-versa* still remains unclear.

Hexosamine biosynthesis pathway is an important nutrient signaling pathway found to be activated in majority of cancers (7, 8). Major metabolites, glucose, glutamine, acetyl CoA, and UTP feed into hexosamine biosynthesis pathway, making this pathway central to sensing the metabolic status of the cells (9). The end product of this pathway, UDP-N acetyl glucosamine (UDP-GlcNAc) is the substrate for branched O- and N-glycosylation (10) but is also used as substrate for posttranslational protein monoglycosylation (O-GlcNAcylation) of hundreds of nuclear, cytoplasmic, and mitochondrial proteins (9, 10). This reaction is catalyzed by a single pair of enzymes, O-GlcNAc-transferase (OGT) which adds the GlcNAc moiety and O-GlcNAcase (OGA) which removes it from target proteins. Therefore, dynamic protein O-GlcNAcylation by the action of OGT/OGA enzymes could be central to relaying information on nutrient status of the cell to various cellular processes including gene expression regulation. In cancers, this mechanism could play an important role in regulating gene expression changes associated with metabolic adaptations (11, 12).

In order to investigate the mechanisms of OGT/OGA and protein O-GlcNAcylation on gene regulation in cancer, we decided to determine the effect of OGT and OGA knockdown in human glioblastoma cells. OGT reduction led to significantly reduced glioblastoma cell proliferation, migration, and invasion. Whole transcriptome analysis after OGT knockdown revealed downregulation of several genes known to be crucial for cell proliferation, tumor invasion, and metastasis. Moreover, we found that many of the genes downregulated due to OGT knockdown are also known to be regulated by BRD4. BRD4 is a member of the bromodomain and extra-terminal domain (BET) protein family and contains two bromodomains at its N-terminal region through which it binds to acetylated histones (13) facilitating the recruitment of transcriptional regulators like positive transcription elongation factor b (P-TEFb) and the Mediator complex (14). BRD4 is also known to promote tumorigenesis by the activation of genes involved in cell proliferation and cell-cycle progression. Likewise, OGT catalyzed O-GlcNAcylation of a variety of transcriptional regulators is fundamentally important in cancer cells (12). This suggests the potential existence of shared transcriptional regulatory mechanisms between OGT and BRD4.

However, our results show that BRD4 and OGT may be acting independently in the process of transcription regulation of a common set of genes. In accordance with this, combined inhibition of OGT and BRD4 led to drastically reduced cell proliferation, migration, and invasion of glioblastoma cells than inhibition of BRD4 or OGT alone indicating that OGT and BRD4 act synergistically in the regulation of genes with significant roles in glioblastoma carcinogenesis and could be applied therapeutically.

## Results

### OGT knockdown leads to reduced glioblastoma cell proliferation

**Figure 1:**
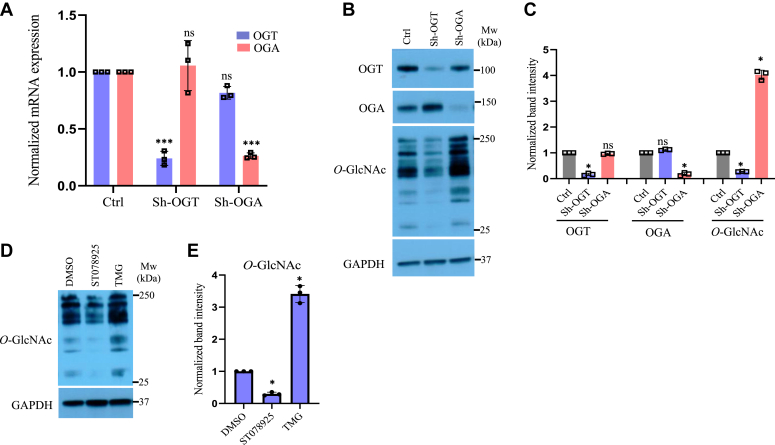
**shRNA mediated OGT and OGA knockdown and their chemical inhibition affects global O-GlcNAc levels in U87 cells.***A*, mRNA expression analysis of OGT and OGA genes in U87 cells transduced with lentiviral vectors carrying shRNAs for OGT and OGA, respectively, compared to empty vector (Ctrl) was performed by real time q-PCR using gene specific primers. *B*, protein expression of OGT and OGA enzymes and total O-GlcNAc levels in U87 cells was analyzed through Western blotting and protein expression of GAPDH was used as loading control. *C*, densitometric quantitation of Western blots from panel B. *D*, U87 cells were treated with OGT inhibitor, ST078925 and OGA inhibitor, thiamet G (TMG) for 72 h before Western blotting for total O-GlcNAc levels. *E*, densitometric quantitation of Western blots from panel D. OGA, O-GlcNAcase; OGT, O-GlcNAc transferase; q-PCR, quantitative PCR.

The expression of OGT is found to be increased in a wide variety of cancers whereas misexpression (both upregulation and downregulation) of OGA is also similarly reported (15). Therefore, in order to understand the role of OGT and OGA in glioblastoma, we depleted the levels of these enzymes in U87 cells by using shRNAs delivered through lentivirus. A significantly reduced mRNA (an average of 76% and 74%, respectively) and protein expression (an average of 80% and 82%, respectively) for both OGT and OGA was achieved with an expected effect on global O-GlcNAc levels which was reduced (73%) with OGT knockdown and elevated (4 fold) with OGA knockdown (Fig. 1, *A*–*C*). Furthermore, pharmacological inhibition of OGT and OGA activity by using known and potent inhibitors, ST078925 and thiamet G (TMG,) respectively, led to reduced (ST078925) and elevated (TMG) global O-GlcNAc levels than dimethylsulfoxide (DMSO) control as observed through Western blotting (Fig. 1, *D* and *E*).

**Figure 2:**
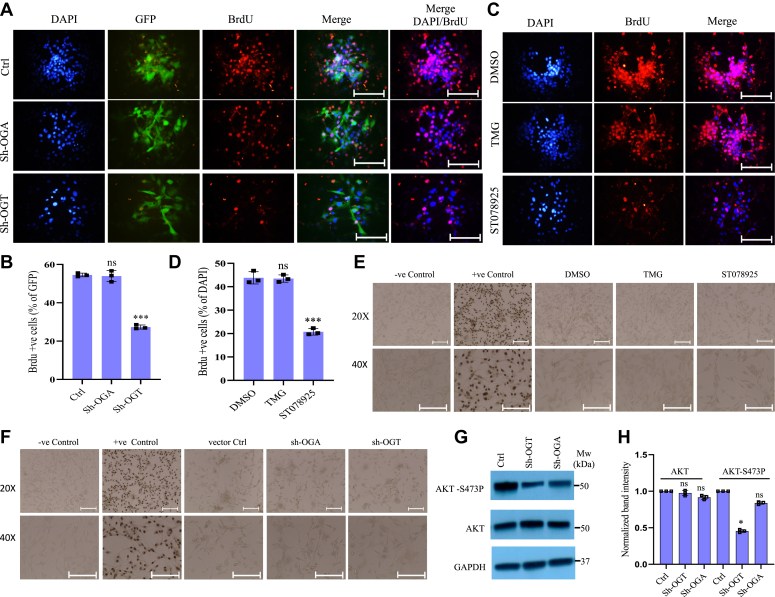
**OGT suppression led to reduced proliferation of glioblastoma cells.***A*, bromodeoxyuridine (BrdU) staining was done to assess cell proliferation in U87 cells transduced with lentiviral vectors carrying shRNAs for OGT and OGA and expressing GFP were compared to Ctrl. *B*, the percentage of BrdU positive cells from panel *A* was calculated using ImageJ software and plotted over GFP positive cells. *C* and *D*, same as panels *A* and *B*, respectively, except U87 cells were treated with OGT inhibitor, ST078925 and OGA inhibitor, TMG for 72 h and the percentage of BrdU positive cells was plotted over percentage of ; positive cells. *E* and *F*, TUNEL staining to assess cytotoxicity in U87 cells treated with OGT inhibitor, ST078925 and OGA inhibitor, TMG (*E*) or transduced with lentiviral vectors carrying shRNAs for OGT and OGA (*F*). *G*, Western blot analysis of AKT and AKT(Ser473P) in U87 cells transduced with lentiviral vectors carrying shRNAs for OGT and OGA compared to Ctrl. GAPDH was used as loading control. *H*, densitometric quantitation of Western blots from panel *G*. The scale bar represents 100 μM. Data represent mean of three biological replicates ± SD. *Asterisks* represent differences being significant (∗*p* < 0.05, ∗∗*p* < 0.01, ∗∗∗*p* < 0.001). ns (not significant). OGA, O-GlcNAcase; OGT, O-GlcNAc transferase; TMG, thiamet G.

Next, we analyzed the effects of OGT and OGA reduction on cell proliferation and cell death. OGT knockdown as well as ST078925 treatment led to significantly reduced cell proliferation as observed through bromodeoxyuridine (BrdU) labeling (Fig. 2, *A*–*D*) whereas no major change was observed for OGA knockdown or TMG treatment as compared to control cells. However, TUNEL assay to check cellular apoptosis did not show any change in either OGT or OGA knockdown or ST078925 as well as TMG treated cells compared to controls (Fig. 2, *E*, *F*, *J*, and *K*). Akt signaling is known to be involved in cell proliferation and cell survival and previous studies have shown an extensive crosstalk between Akt pathway and O-GlcNAc signaling (16, 17). When checked, we found the levels of AKT-S473P significantly reduced in OGT depleted cells whereas OGA depletion did not show a significant change. The expression of total AKT did not change either (Fig. 2, *G* and *H*).

These results suggest that reduction of OGT or inhibiting its activity negatively affects cell proliferation without a major effect on apoptosis which may have been caused due to reduced Akt signaling. Whereas, OGA reduction or inhibition does not affect cell proliferation and/or cell death.

### OGT is required for the expression of glioblastoma promoting genes in U87 cells

Protein O-GlcNAcylation catalyzed by OGT occurs on hundreds of nucleocytoplasmic and mitochondrial proteins in both normal and diseased cells (18, 19). Interestingly, many of OGT targets are gene specific transcription factors (TFs) and chromatin regulators (19, 20) suggesting an important role of OGT in transcriptional regulation through O-GlcNAcylation of these proteins. OGT can directly associate with chromatin on promoters and regulate gene expression through O-GlcNAcylation of histone proteins or modulating other histone modifications (12). In addition, previous studies have clearly shown the involvement of both OGT and OGA enzymes in the regulation of basal Pol II transcription machinery through O-GlcNAcylation of various proteins including RNA Pol II C-terminal domain (CTD) (21).

**Figure 3:**
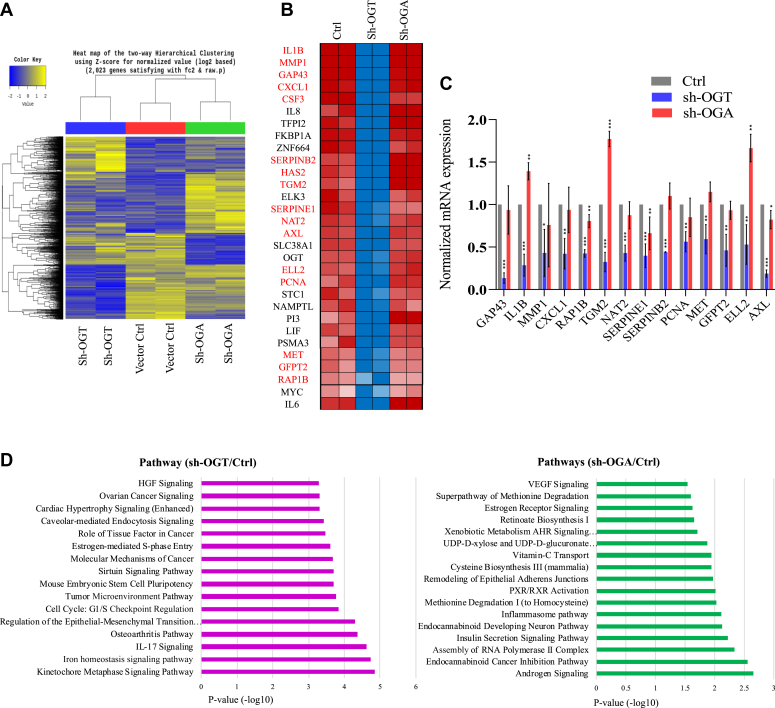
**OGT suppression downregulates genes known for glioblastoma progression.***A*, hierarchical clustering analysis and corresponding heat map of differentially expressed genes in sh-OGT and sh-OGA and empty vector (Ctrl) U87 cells. *B*, heat map of top genes downregulated due to OGT depletion (sh-OGT) compared to Ctrl and sh-OGA U87 cells. *C*, real time PCR validation of top 14 genes (highlighted in *red* in panel *B*) downregulated due to OGT depletion using gene-specific primers. Data represent mean of three biological replicates ± SD. *Asterisks* represent differences being significant (∗*p* < 0.05, ∗∗*p* < 0.01, ∗∗∗*p* < 0.001). *D*, ingenuity pathway analysis was performed on the list of genes significantly affected (*p*-value < 0.5) in sh-OGT and sh-OGA cells compared to Ctrl. OGT, OGA, O-GlcNAcase; O-GlcNAc transferase.

Therefore, we analyzed the effect of OGT and OGA reduction on transcription genome-wide through RNA-Seq in U87 cells. OGT knockdown led to downregulation of 596 genes whereas 499 genes were upregulated more than two folds whereas OGA knockdown resulted in 487 genes being downregulated and 699 genes were upregulated (Figs. 3*A* and S1). Further focus on differentially expressed genes (DEGs) in OGT knockdown cells revealed decreased expression of several genes involved in glioblastoma cell proliferation, invasion and immune modulation such as IL1B, MMP1, GAP43, CXCL1, PCNA, LIF, MYC, IL6 among others (Fig. 3*B*). Quantitative real time PCR validation of top 14 genes downregulated due to OGT reduction confirmed significantly reduced expression due to OGT knockdown whereas OGA reduction did not affect expression of most of these genes except IL1B, TGM2 and ELL2 showed significant upregulation whereas RAP1B, SERPINE1, and AXL showed subtle but significantly reduced expression (Fig. 3*C*). This suggests that OGT and possibly OGA through O-GlcNAcylation may mediate glioma progression through transcriptional regulation of these genes.

Moreover, pathway analysis on the list of DEGs in OGT knockdown cells showed various cell division and cell cycle pathways, regulation of epithelial to mesenchymal transition, and tumor microenvironment pathways enriched among others (Fig. 3*D*). Whereas, OGA reduced cells showed enrichment of various hormone signaling pathways such as androgen signaling, insulin secretion and signaling, estrogen receptor signaling, and amino acid, methionine, and cysteine synthesis/degradation pathways among others (Fig. 3*D*). These results further indicate the involvement of OGT in carcinogenesis whereas OGA could be mainly involved in metabolic pathways.

Since, OGT is known to functionally regulate many TFs through O-GlcNAcylation, we checked whether genes downregulated due to OGT reduction are commonly regulated through specific TFs. Thus, we used web-based tools of TF enrichment analysis on the list of genes significantly downregulated due to OGT knockdown. We found BRD4 as the top transcriptional regulator enriched on downregulated genes along with several other known gene specific TFs such as P53, FOSL1/2, MYC, YAP1, and JUN among others (Fig. S2, *A* and *B*). Notably, the expression of MYC was also reduced due to OGT knockdown (Fig. 3*B*). BRD4 is a transcription cofactor which interacts with several sequence-specific TFs including those listed above and binds to chromatin through recognition of acetylated histone proteins (22, 23). BRD4 also acts as a transcription elongation factor where it helps in the recruitment of positive transcription elongation factor b (pTEFb) on promoters (24). This raises the possibility that BRD4 may commonly regulate transcription of OGT dependent genes through interacting with several of these enriched TFs.

### Effect of OGT reduction on the recruitment of BRD4 on target gene promoters

**Figure 4:**
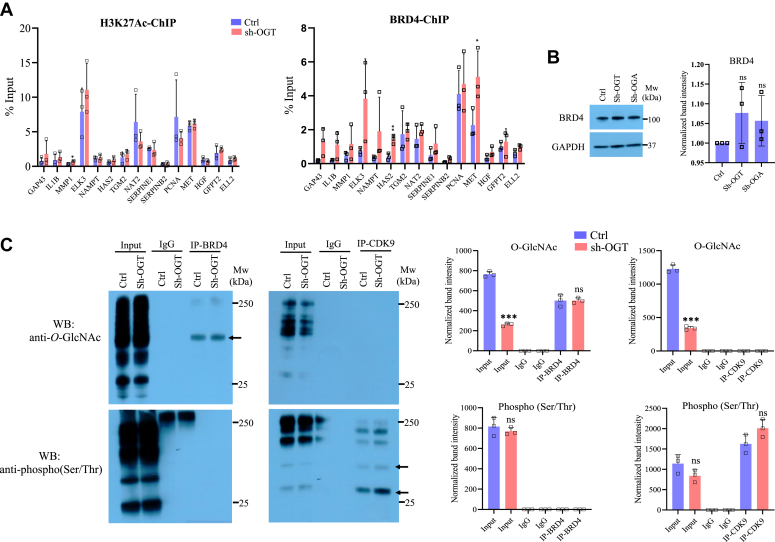
**OGT suppression does not affect BRD4 expression or its O-GlcNAcylation.***A*, ChIP assay was done to analyze the enrichment of histone, H3K27Ac and BRD4 at the promoter (−500 to +500 bp of TSS) of the indicated genes using ChIP grade antibodies in empty vector (Ctrl) and sh-OGT U87 cells. *B*, Western blot analysis of BRD4 in Ctrl, sh-OGT and sh-OGA U87 cells (*right panel*) and densitometric quantitation (*left panel*) of Western blots from *right panel*. GAPDH was used as loading control. *C*, co-immunoprecipitation (Co-IP) was performed on cell lysates from Ctrl and sh-OGT U87 cells using anti-BRD4 or anti-CDK9 antibodies followed by Western blotting of IP’d samples using anti-O-GlcNAc and anti-phospho(Ser/Thr) antibodies. IP with IgG was used as control. Input is the total cell lysate (*left panel*) and densitometric quantitation (*right panel*) of Western blots from *left panel*. Data represent the mean of three biological replicates ± SD. Statistical analyses (unpaired student’s *t* test) were made between control and treated samples using GraphPad Prism Software. *Asterisks* represent differences being significant (∗*p* < 0.05, ∗∗*p* < 0.01). ChIP, chromatin immunoprecipitation; IgG, immunoglobulin G; IP, immunoprecipitation; OGA, O-GlcNAcase; OGT, O-GlcNAc transferase; TSS, transcription start site.

Next, we sought to understand the interaction between OGT and BRD4, given its role in the regulation of OGT-dependent genes (Fig. S2*A*). OGT knockdown did not change protein expression levels of BRD4 in U87 cells as observed in Western blotting (Fig. 4*B*). Previous studies have found BRD4 to be phosphorylated as well as O-GlcNAcylated. In order to find out whether OGT reduction could have affected BRD4 O-GlcNAcylation and/or its phosphorylation, we immunoprecipitated BRD4 and probed with anti-O-GlcNAc and anti-phospho-Ser/Thr antibodies. BRD4 was not phosphorylated in these cells whereas we found it to be O-GlcNAcylated but surprisingly OGT knockdown resulted in no major change in BRD4 O-GlcNAcylation levels. (Fig. 4*C*). To confirm this result, we treated U87 cells with both OGT and OGA inhibitors, ST078925 and TMG, respectively, followed by immunoprecipitation of BRD4 and probed with anti-O-GlcNAc antibody. The results again showed no change in BRD4 O-GlcNAc levels due to either OGT or OGA inhibition (Fig. S3, *A* and *B*). Protein expression of BRD4 also did not change using two different inhibitors of OGT (Fig. S3, *C* and *D*).

Since BRD4 is recruited to promoters due to recognition of acetylated histone through its bromodomain, we analyzed enrichment of histone H3K27Ac as well as BRD4 through chromatin immunoprecipitation (ChIP) of top fifteen genes downregulated due to OGT knockdown. Promoter regions 500 bp upstream and downstream of transcription start site were analyzed. We did not find a change in the levels of H3K27Ac on the promoters of these genes except for a subtle increase on MMP1 in OGT dependent genes analyzed; however, the levels of BRD4 was higher (although not statistically significant) on many of these genes in OGT depleted cells compared to control (Fig. 4*A*).

These results led us to conclude that although BRD4 is O-GlcNAcylated in these glioblastoma cells, OGT reduction or its inhibition neither affected the levels of this modification nor the protein levels of BRD4. However, since knockdown of OGT in these experiments is not complete (80% reduced protein expression), the remaining 20% of OGT might be able to keep some of the targets O-GlcNAcylated especially those which have high affinity for OGT or with tight O-GlcNAc cycling rates and BRD4 could be one such OGT target. In accordance with these results, there was either no major change or a subtle increase in the recruitment of BRD4 on OGT dependent gene promoters whereas H3K27Ac levels remained unchanged due to OGT reduction on most of genes.

### General transcription machinery remains unperturbed except for Pol II-Ser2P due to OGT reduction on most of OGT dependent genes

**Figure 5:**
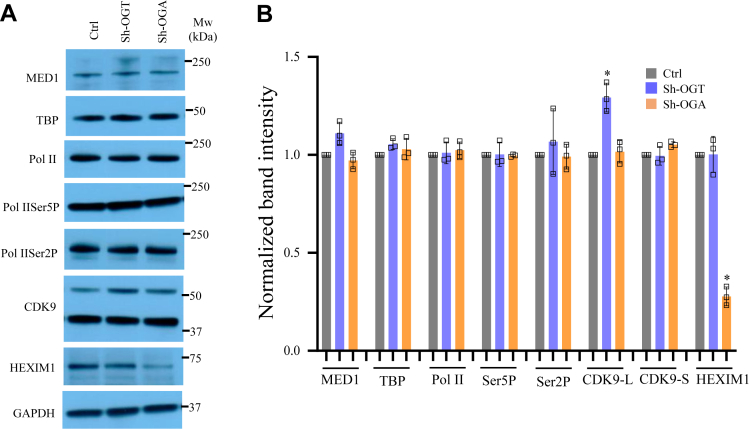

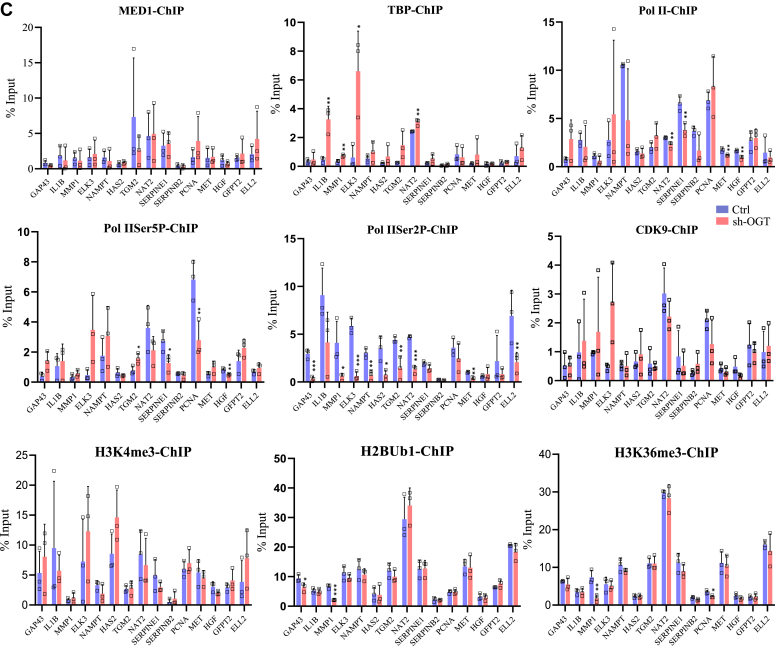

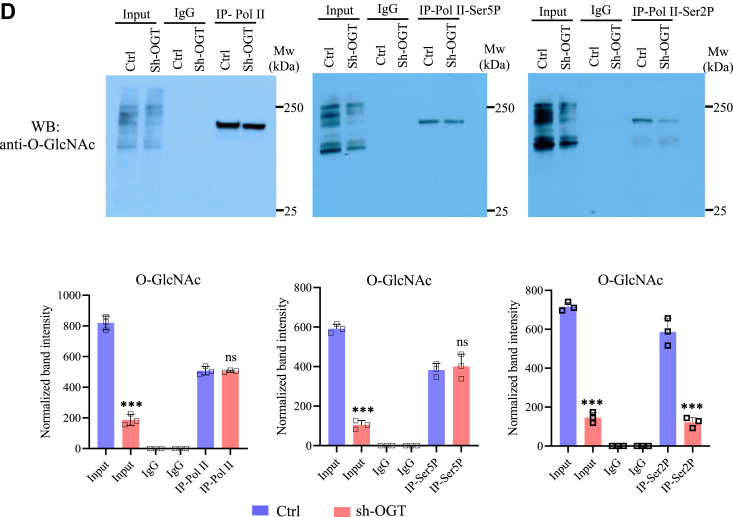
**Effect of OGT and OGA suppression on the formation of transcriptional preinitiation and elongation complexes.***A*, Western blot analysis of MED1, TBP, RNA Pol II, Pol II-Ser5P, Pol II-Ser2P, CDK9, and HEXIM1 in Ctrl, sh-OGT, and sh-OGA U87 cells. *B*, densitometric quantitation of Western blots from panel *A*. GAPDH (same as in Fig. 2*G*) was used as the loading control. *C*, ChIP assay was done to analyze the enrichment of the same proteins as in panel *A* and histone, H3K4me3, H2BK120Ub1, and H3K36me3 at the promoter (−500 to +500 bp of TSS) of the indicated genes using ChIP grade antibodies in empty vector (Ctrl) and sh-OGT U87 cells. *D*, co-immunoprecipitation (Co-IP) was done on cell lysates from Ctrl and sh-OGT U87 cells using anti-Pol II, anti-Pol II-Ser5P, and anti-Pol II-Ser2P antibodies followed by Western blotting of IP’d samples using anti-O-GlcNAc antibody. IP with IgG was used as control. Input is the total cell lysate (*top panel*) and densitometric quantitation of Western blots (*bottom panel*). Data represent mean of three biological replicates ± SD. Statistical analyses (unpaired student’s *t* test) were made between control and treated samples using GraphPad Prism Software. *Asterisks* represent differences being significant (∗*p* < 0.05, ∗∗*p* < 0.01, ∗∗∗*p* < 0.001). ChIP, chromatin immunoprecipitation; IgG, immunoglobulin G; IP, immunoprecipitation; OGA, O-GlcNAcase; OGT, O-GlcNAc transferase; TSS, transcription start site.

In addition to regulating gene-specific TFs and chromatin regulators through O-GlcNAcylation, previous studies have reported the role of OGT and protein O-GlcNAcylation in the direct regulation of transcription through its regulation of basal transcription machinery (25, 26). In order to find out whether OGT knockdown could have directly affected basal transcription machinery and thus affecting transcription of its target genes, we checked protein expression of TBP, MED1, unmodified Pol II, Pol II-Ser5P (enriched upon transcription initiation) and Pol II-Ser2P (enriched upon active transcription elongation), CDK9 and HEXIM1 in OGT and OGA knockdown cells compared to control. Both OGT and OGA reduction did not affect expression of any of these TFs as observed through Western blotting except for CDK9 and HEXIM1 (Fig. 5, *A* and *B*). Mammalian cells express two different isoforms of CDK9, Cdk9-S(42 kDa), and Cdk9-L(55 kDa) (27), and we noticed a subtle but significant increase in Cdk9-L(55 kDa) in OGT knockdown cells. In addition, HEXIM1 which forms an inhibitory complex with CDK9 was reduced significantly upon OGA reduction suggesting that increased O-GlcNAc levels may negatively affect HEXIM1 expression (Fig. 5, *A* and *B*).

Next, we analyzed the binding of TBP, MED1, CDK9, Pol II, Pol II-Ser5P, and Pol II-Ser2P on the promoter region (500 bp upstream/downstream of transcription start site) through ChIP of the same top fifteen most downregulated genes due to OGT reduction as above (Fig. 4*A*). No major change was observed on the recruitment of MED1 at the promoters of all gene tested in sh-OGT cells compared to control. Whereas, TBP recruitment was higher on many of these genes in sh-OGT cells with the increase being statistically significant for IL1B, MMP1, ELK3, and NAT2. Pol II binding did not change due to OGT knockdown on most genes except for NAT2, SERPINE1, MET, and HGF which showed subtle but significantly reduced levels. Pol II-Ser5P was also reduced on SERPINE1, PCNA, and HGF, and an increase was noted on TGM2 (Fig. 5*C*). Interestingly, however, Pol II-Ser2P levels were significantly reduced on many of the genes analyzed due to OGT knockdown. Ser2 of Pol II is phosphorylated mainly by CDK9 which is a component of pTEFb complex upon active transcription elongation. CDK9 recruitment, however, did not change upon OGT reduction on any of the genes analyzed here except for ELK3 (Fig. 5*C*). We also analyzed histone marks H3K36me3 and H2BK120Ub1 which are enriched on gene bodies during active transcription elongation and H3K4me4, a mark of active transcription. Surprisingly, we did not see any major change in the levels of any of these histone marks on all of the fifteen genes analyzed here except for GAP43 and MMP1 (H2BK120Ub1) and MMP1 and PCNA (H3K36me3) showing reduced enrichment (Fig. 5*C*). In addition, we also analyzed binding of OGT on these promoters. However, we did not see OGT recruitment above background levels on these genes (data not shown) suggesting that any role of OGT at these steps of transcription cycle may be due to OGT mediated O-GlcNAcylation of effector proteins rather than its direct promoter association. Prior research has identified O-GlcNAcylation of RNA Pol II CTD during specific stages of the transcription cycle, alongside phosphorylation (26, 28). Therefore, we conducted co-immunoprecipitation (Co-IP) experiments for Pol II, Pol II-Ser5P, and Pol II-Ser2P, followed by probing with anti-O-GlcNAc antibody. The aim was to investigate whether O-GlcNAcylation occurs on Pol II in U87 cells and the impact of OGT knockdown on this if any.

Remarkably, our findings revealed that all three forms of Pol II undergo O-GlcNAcylation. Interestingly, OGT knockdown resulted in a notable decrease in O-GlcNAc levels specifically on Pol II-Ser2P, while O-GlcNAc levels on Pol II and Pol II-Ser5P showed minimal changes (Fig. 5*D*). This outcome strongly indicates that although Pol II is O-GlcNAcylated in all stages of transcription cycle (analyzed here through Pol II phosphorylation), this modification is particularly dynamic on actively elongating Pol II. This dynamic O-GlcNAcylation might play a crucial role in the chromatin association of Pol II during transcription elongation. Consequently, the diminished O-GlcNAcylation of Pol II-Ser2P due to OGT knockdown could potentially account for the decreased binding of Pol II-Ser2P to OGT target genes, as we have observed through ChIP (Fig. 5*C*).

In summary, these findings collectively lead us to deduce that OGT catalyzed O-GlcNAcylation of elongating Pol II holds a vital role in its chromatin association with OGT target genes. Consequently, the reduction of OGT levels could feasibly contribute to a decline in transcription elongation process. A subtle but significant reduction of Pol II and Pol II-Ser5P observed on some of the genes through ChIP may indicate reduced transcription initiation as well. However, since these changes were not commonly observed on all genes analyzed here, this suggests that transcription machinery remained essentially unperturbed due to OGT knockdown.

### Combined inhibition of OGT and BRD4 synergistically reduced glioblastoma cell proliferation, migration, and invasion

**Figure 6:**
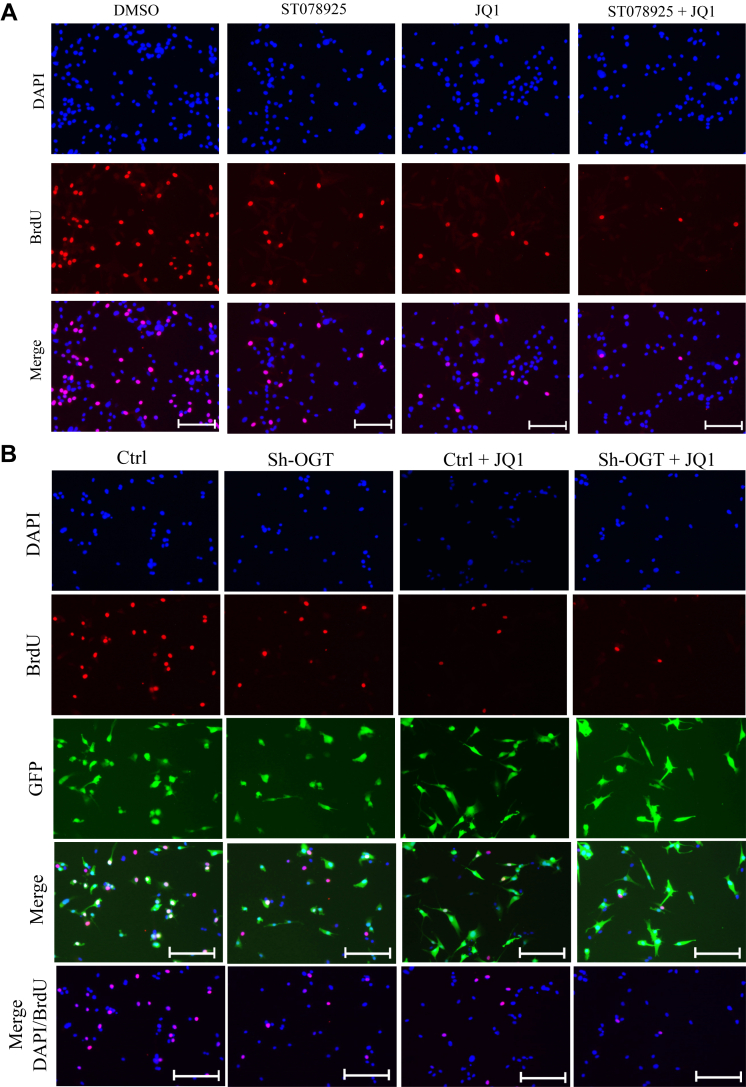

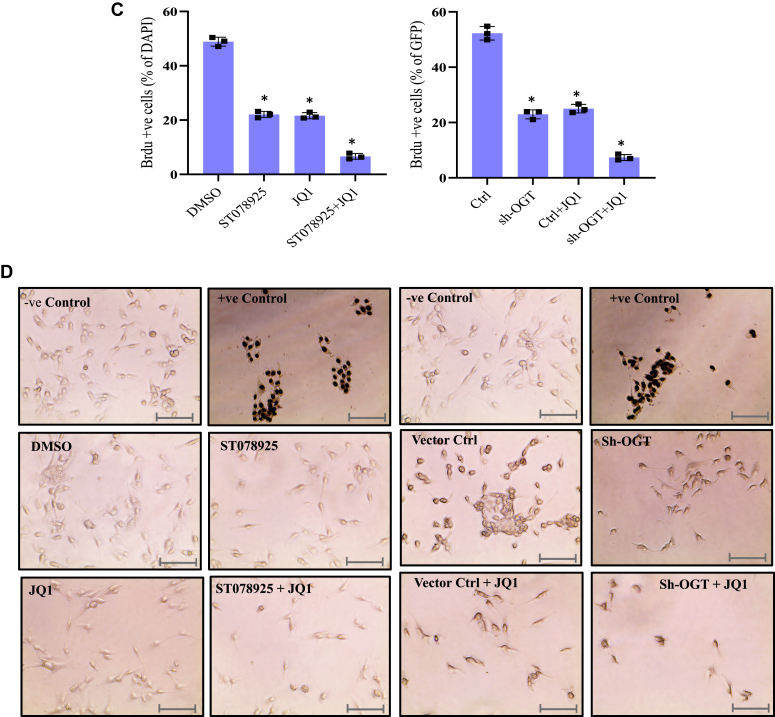

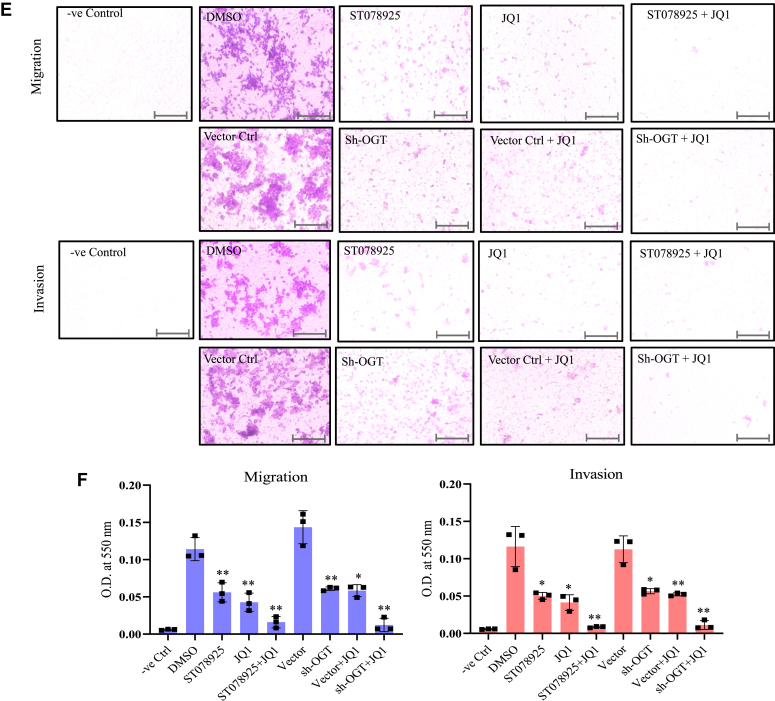
**Combined inhibition of OGT and BRD4 synergistically affects U87 cell proliferation, migration, and invasion.***A*, BrdU staining was done to assess cell proliferation of U87 cells treated with solvent (DMSO), ST078925, JQ1 and ST078925 + JQ1. *B*, BrdU staining was done to assess cell proliferation of empty vector (Ctrl), sh-OGT, Ctrl treated with JQ1 (Ctrl +JQ1) and sh-OGT treated with JQ1 (sh-OGT + JQ1) U87 cells expressing GFP. *C*, the percentage of BrdU positive cells from panel *A* was plotted over percentage of DAPI positive cells (*left graph*) and from panel *B* was plotted over GFP positive cells (*right graph*) using ImageJ software. *D*, TUNEL staining to assess cytotoxicity in U87 cells with treatments same as in panel *A* (*left side*) and panel *B* (*right side*). *E*, cell migration and invasion assay was performed on U87 cells treated with same combination of vectors and chemical inhibitors as described in panels *A* and *B* in a transwell plate, and cells were stained with crystal violet solution. *F*, absorbance of solubilized crystal violet dye from corresponding cells of panel *E* was measured at 595 nm. The scale bar  represents 100 μM. Data represent mean of three biological replicates ± SD. Statistical analyses (unpaired student’s *t* test) were made between control and treated samples using GraphPad Prism Software. *Asterisks* represent differences being significant (∗*p* < 0.05, ∗∗*p* < 0.01). BrdU, bromodeoxyuridine; DAPI, 4′,6-diamidino-2-phenylindole; DMSO, dimethylsulfoxide; OGT, O-GlcNAc transferase.

Numerous studies in the past have shown BRD4 to be dysregulated in various types of cancers including glioblastoma (29, 30), and BRD4 inhibitors are already in clinical trials as glioblastoma therapy (31, 32, 33). Similarly, OGT inhibition is reported to reduce cancer cell proliferation and metastasis in several types of cancers (15, 34, 35). The main function of BRD4 in these cancers is acting as a positive regulator of transcription of target genes. As we found the expression of BRD4 or its recruitment on OGT target genes remains unaffected due to OGT reduction, we postulated that BRD4 inhibition along with OGT reduction may have a synergistic effect on mRNA expression of target genes, thus affecting glioblastoma cells. In order to test this, we performed BrdU labeling to assess cell proliferation after indicated treatments of U87 cells. As shown in Figure 6*A*, U87 cells treated with solvent (DMSO) only showed around 50% cells to be BrdU positive. However, treatment with either ST078925 or JQ1 (BRD4 inhibitor) led to reduction of % BrdU positive cells to more than half of the control group. Interestingly, a more profound decrease in the number of proliferating cells was observed when U87 cells were treated with a combination of both ST078925 and JQ1 as compared to single treatments thus showing synergistic effect (Fig. 6, *A* and *C*).

Next, U87 cells were infected with lentiviral particles expressing shRNA either for OGT or empty vector backbone (Ctrl) as well as treated with JQ1 in Ctrl and sh-OGT cells. The results showed the same trends as described above for chemical treatments. OGT knockdown using shRNA as well as JQ1 treatment of vector only infected cells showed more than 50% reduction in % BrdU positive cells as compared to control group. A synergistic effect of OGT knockdown and JQ1 treatment was observed as %BrdU positive cells were reduced to less than 10% in the combined treatments (Fig. 6*C*). In contrast to cell proliferation, apoptosis was not affected by any of these treatments as evidenced by TUNEL assay (Fig. 6*D*).

Among several methods used to study migratory ability of cancer cells, transwell cell migration assay is most direct as well as readily adaptable to quantitative analysis. For cell invasion experiments, the same transwells used for migration were first coated with Matrigel before seeding the cells. Complete growth media (Dulbecco's modified Eagle's medium [DMEM]/F12 + 10% fetal bovine serum [FBS]) supplemented with 250 ng/ml of epidermal growth factor was used as chemoattractant in the lower compartment of the transwell chamber and U87 cells following different treatments as described above were seeded on the upper chamber followed by incubation for 16 h. For negative control, only DMEM/F12 media was used in the lower chamber. Transwell inserts were scrapped from above to remove the nonmigrating cells and stained by using crystal violet. For both migration and invasion analysis, marked purple color staining was observed for DMSO treated U87 cells and no staining observed for negative control cells. The staining was significantly reduced following treatment of cells with either ST078925 or JQ1 for both migration and invasion analysis. Combined treatment of ST078925 and JQ1 resulted in further decreased staining response as compared to single treatments showing synergistic behavior (Fig. 6*E*).

Similarly, OGT knockdown resulted in marked decrease of migration and invasion of cells as compared to empty vector infected Ctrl cells which showed intense purple staining. A similar decrease in staining was observed when empty vector infected Ctrl cells were treated with JQ1. Finally, the staining response was further reduced when OGT shRNA expressing U87 cells were treated with JQ1 (Fig. 6*E*). Quantitative analysis of the staining response corroborated well with the staining analysis described above (Fig. 6*F*).

## Discussion

Protein O-GlcNAcylation levels are found to be upregulated in most of the cancers (36). Therefore, targeting O-GlcNAcylation through chemical inhibition of OGT and OGA enzymes has been proposed as an effective strategy for cancer treatment. However, due to their pleiotropic effects on cellular processes, it is imperative to identify their mechanisms of actions in specific cancer types. Here, we have investigated the role of OGT and OGA enzymes in glioblastoma cells through their chemical inhibition and gene knockdown. We found that OGT knockdown, in particular led to reduced expression of many genes with prominent roles in glioblastoma progression and cancer cell invasion. The top most affected genes included GAP43 which has recently been shown to play a key role in glioma invasiveness through tumor microtube formation (37) and MMP1 which facilitates tumor microtubes' infiltration throughout the brain (38, 39). These studies also suggest that downregulation of MMPs or GAP43 is sufficient to prevent glioblastoma progression. CXCL1 and CXCL2 are also among the top genes that were downregulated due to OGT knockdown. Both of these genes are found to be overexpressed in aggressive glioblastomas where they promote immune suppression of glioblastoma and accelerate tumor progression (40). In addition, expression of interleukin genes IL1B, IL8, and IL6 was significantly reduced upon OGT knockdown. Previous studies have shown these interleukins to be significantly upregulated in glioblastoma multiforme cell lines and tissue samples and may result in cancer-induced inflammation (41, 42). Another OGT target gene with a significant role in glioblastoma tumorigenesis is nicotinamide phosphoribosyltransferase (NAMPT), which is a rate-limiting enzyme of the salvage pathway to regenerate adenine dinucleotide (NAD). Studies have reported that NAMPT overexpression led to increased glioma stemness (43) and glioblastoma multiforme patients with higher levels of NAMPT expression had poor prognosis than those with lower expression (44). Interestingly, knockdown of NAMPT expression in the same glioblastoma cells (U87) as used in our study led to decreased cell proliferation, migration, and invasion and reduced tumor growth in-vivo (44).

Transcriptional downregulation of these specific glioblastoma associated genes due to OGT reduction indicates that OGT may be required for glioblastoma maintenance and progression which is also supported by previous reports for a variety of other cancers (45). It is also recognized that O-GlcNAcylation of many transcriptional regulators catalyzed by OGT contribute to gene expression regulation (46). Searching for such transcriptional regulators for the genes which showed reduced expression due to OGT reduction in this study, we found BRD4 as a common regulator of many of these genes. BRD4 is a bromodomain containing protein that acts as a positive regulator of transcription initiation through its interaction with acetylated histone proteins on chromatin and gene specific transcription activator proteins at promoter regions (22). BRD4 is found to be phosphorylated leading to its stability and tumor-promoting transcriptional program (47). Interestingly, BRD4 is also found to be O-GlcNAcylated (48) although the role of BRD4 O-GlcNAcylation in transcription is not studied. Based on these evidences, we hypothesized that BRD4 could be O-GlcNAcylated by OGT in glioblastoma which may regulate its function in transcription regulation of target genes. Co-IP experiments in this study proved BRD4 to be O-GlcNAcylated but not phosphorylated at Ser/Thr residues although, Tyr phosphorylation of BRD4 cannot be ruled out. Surprisingly, OGT knockdown or chemical inhibition did not affect BRD4 O-GlcNAcylation. Therefore, function of O-GlcNAcylated BRD4 in transcription of OGT target genes, if any may not have been affected due to OGT reduction. Most important role of BRD4 in the process of transcription regulation is to directly bind to cyclinT1 and Cdk9, the constituents of pTEFb complex, thus regulating transcription elongation (49). Phosphorylation of Ser2 of Pol II CTD by CDK9 is a crucial step in the transition from initiation to active elongation phase of mRNA transcription (50). However, a large percentage of CDK9 is associated with an inhibitory complex comprised of 7SK snRNA and MePCE, LARP7, and HEXIM1/2 proteins (51). In its active form, CDK9 is phosphorylated on the threonine 186 residue (Thr186). In addition, CDK9 is found to be O-GlcNAcylated as well (48). However, we did not find CDK9 to be O-GlcNAcylated in U87 cells whereas it was found to be phosphorylated as expected. Although OGT depletion did not affect CDK9 phosphorylation, we found a subtle but significant increase in the expression of large isoform (55 Kda) of CDK9 through Western blotting. Small isoform of CDK9 is well characterized for its function, whereas a previous report suggests 55 Kda isoform to be predominant in rat quiescent hepatocytes whereas 42 Kda isoform was induced upon cell cycle entry (27). However, there are no further information on the isoform specific roles of CDK9 in cell division. We believe, an increase in large isoform upon OGT depletion may indicate cellular quiescence as we have observed significantly reduced cell proliferation due to OGT depletion. This further leads to the assumption on the role of large CDK9 isoform in Pol II-Ser2P and could be related to reduced Pol II-Ser2P observed on OGT target genes. Furthermore, our findings also revealed that all three forms of Pol II analyzed in this study, unmodified Pol II, Pol II-Ser5P, and Pol II-Ser2P undergo O-GlcNAcylation whereas OGT knockdown significantly reduced O-GlcNAc levels of only Pol II-Ser2P. This result clearly suggests that O-GlcNAcylation is highly dynamic on actively elongating Pol II and might be required for its chromatin association during transcription elongation. Consequently, decreased O-GlcNAcylation on Pol II-Ser2P due to OGT knockdown could plausibly account for the decreased binding of Pol II-Ser2P to OGT-targeted genes, corroborated by our observations from ChIP assays.

It is also important to note that ChIP analysis done for general transcription initiation factors, TBP, MED1, Pol II, and Pol II-Ser5P and elongation factors BRD4, CDK9, and transcription initiation and elongation related histone modifications did not show a major change on many of the promoters of the top 15 OGT-dependent genes. This outcome further confirms that reduced transcription of these genes, due to OGT reduction may not have resulted from defects in transcription initiation rather result from reduced transcription elongation.

Noticeably, we found that OGT knockdown led to significantly reduced proliferation of U87 cells. An extensive crosstalk between O-GlcNAc and Akt signaling pathway is reported through previous studies (16, 17). OGT was required for Akt driven proliferation of, for example, pancreatic β-cells in previous studies (52) and increased pAkt expression is associated with poor prognosis in glioblastoma patients (53). We found that OGT reduction significantly reduced AKT-Ser473P suggesting that OGT may regulate glioblastoma cell proliferation through Akt pathway.

Finally, as we recognized that OGT reduction or chemical inhibition did not affect expression and O-GlcNAc levels of BRD4 and as BRD4 is known to regulate transcription initiation/elongation steps, we assume that OGT regulates transcription independently of BRD4. Thus, we believed that combined inhibition of OGT and BRD4 may have synergetic effect on glioblastoma cells. Indeed, combined inhibition resulted in more drastic decrease in U87 cell proliferation, invasion, and migration.

In conclusion, our results suggest that OGT and BRD4 regulate a common set of genes involved in glioblastoma proliferation, invasion, and migration but act independently in terms of their role in transcriptional regulation. Thus, combined inhibition of OGT and BRD4 could be utilized as a therapeutic strategy for highly efficient glioblastoma targeting than either protein alone.

## Experimental procedures

### Cell culture and treatments

All chemicals for cell culture were purchased from Gibco unless otherwise stated. For lentivirus production, HEK293T cells were grown using DMEM, 10% FBS and 1× penicillin/streptomycin (P/S). U87 cells were routinely cultured in DMEM/F12 medium supplemented with 10% FBS, 1× L-glutamine, and 1× P/S. The cells were treated with 40 μM of TMG, 80 μM of ST078925, 40 μM of JQ1 and a combination of 80 μM of ST078925 and 40 μM of JQ1 for 72 h before analysis. Cells treated with DMSO were used as controls.

### shRNA-lentiviral production and infections

shRNA oligos were annealed and cloned into the HpaI and XhoI digested pLL3.7 vector which contained a GFP marker to monitor virus production and infection. The sequences of shRNAs for OGT and OGA genes were obtained from previous publications (54, 55, 56). Lentivirus particles were generated by cotransfecting shRNA containing pLL3.7 or uncut backbone pLL3.7 vector (as negative control) along with packaging plasmids (psPAX2 and pMD2G) in HEK293T cells by using PEI (Sigma-Aldrich) as described in detail previously (57). The viral supernatants were collected 48 and 72 h posttransfection, clarified by low-speed centrifugation and filtration using 0.45 μm low protein binding polyethersulfone membrane filters (Thermo Fisher Scientific). The viral supernatants were stored at −80 °C in single use aliquots to avoid freeze/thaw cycles. U87 cells were infected twice over a period of 48 h with lentiviral particles in the presence of 8 μg/ml polybrene and cells analyzed for expression of GFP. Cells were harvested at 72 h for analysis postinfection.

### Western blot analysis

Monolayer cells were washed with PBS and directly lysed in 1 ml/10 cm^2^ of 1 × RIPA lysis buffer (Cat. no. 9806; CST) containing 1% SDS and 1× Halt protease and phosphatase inhibitor cocktail (Cat. no. 1861284; Thermo Fisher Scientific) and incubated on ice for 10 to 15 min followed by centrifugation at 14,000 rpm for 10 min at 4 °C. Supernatants were collected and quantified using Pierce BCA Protein Assay Kit (Cat. no. 23225; Thermo Fisher Scientific) following manufacturer’s recommendations. For each sample, quantities were adjusted to 2 mg/ml using Nupage 4 × loading dye sample buffer (NP0007; Life Technologies) and protein samples were denatured at 70 °C for 10 min. Equal amount of proteins were loaded and resolved using SDS-PAGE gels followed by transfer to polyvinylidene difluoride membrane (Cat. no. 88518; Thermo Fisher Scientific) at a constant voltage of 100 V for 1 h at 4 °C. After transfer, the membranes were blocked and incubated in primary antibody dilutions at 4 °C for overnight. Next day, membranes were extensively washed using phosphate-buffered saline with Tween 20 and incubated in horseradish peroxidase-conjugated appropriate secondary antibodies for 1 h at room temperature followed by phosphate-buffered saline with Tween 20 washing. Finally, protein bands were visualized using super signal West Pico chemiluminescent substrate (Cat. no. 34080; Thermo Fisher Scientific) and developed using X-ray films. The blots were quantitated using ImageJ software (https://ij.imjoy.io/). The information on antibodies is provided in the list of antibodies in Table S1.

### BrdU incorporation assay

For these experiments, cells were cultured on P4 well plates and treated accordingly. Following treatments, BrdU (Tocris) was added to media at final concentration of 50 μM to the cells and incubated for 1 h. Cells were washed with PBS and fixed using 4% paraformaldehyde for 15 min at room temperature followed by permeabilization using 0.3% Triton X-100. Cells were incubated with 2N HCl for 15 min at room temperature to denature DNA followed by incubation in Na-borate buffer for 15 min at room temperature. The cells were then blocked for nonspecific binding by using 2% bovine serum albumin (BSA) in PBS for 1 h at room temperature and incubated with anti-BrdU antibody (Abclonal) diluted in 1% BSA overnight at 4 °C. Next day, cells were washed with PBS and incubated with appropriate Alexa Flour–conjugated secondary antibody diluted in 1% BSA for 1 h at room temperature. Finally, the samples were washed with PBS, counterstained with 4′,6-Diamidino-2-Phenylindole, Dihydrochloride (Millipore, CAS 28718-90-3) and imaged using fluorescence microscope (Zeiss, Axiovert).

### RNA sequencing and data analysis

Total RNA was isolated using MasterPure RNA isolation Kit (Epicenter; MCR 85102) following manufacturer’s recommendations. An aliquot equivalent to 1 μg of total RNA was used for each sample. The rRNAs were removed by using NEBNext rRNA Depletion Kit (E7400L) and purified using AMPure XP magnetic beads. Following rRNA removal, the samples were subjected to library preparation using NEBNext Ultra II RNA Library Prep Kit (E7775) using manufacturer’s protocol. The libraries were amplified using NEBNext Multiplex Oligos (E6440S) and each sample was given a unique combination of i5 and i7 index primers for multiplexing. Finally, the samples were purified using AMPure XP beads and subjected to quality check on Bioanalyzer and sequencing.

Sequencing was performed at Macrogen Inc, on Illumina Next-Generation Sequencer (HiSeqXten), followed by bioinformatics analysis. DESeq2 tool was used to identify DEGs between sh-OGT and sh-OGA cells compared to vector control. DEGs with a 2-fold or more change with a *p*-value of ≤0.5 were considered significant. Gene set enrichment analysis was done using Qiagen's Ingenuity Pathway Analysis software (IPA, QIAGEN Redwood City, www.qiagen.com/ingenuity) using “Canonical pathways”, “Diseases &Functions” and “Upstream Regulators” options. Expression heatmaps were generated using DESeq2 normalized count values. Hierarchical clustering was performed using Euclidean distance to visualize the expression of genes across three samples (Ctrl, sh-OGT, and sh-OGA) that were significant in at least one sample type with false discovery rate < 5%.

### cDNA synthesis and real time PCR analysis

Total RNA was isolated (as described above) and 1 μg was subjected to complementary DNA (cDNA) synthesis using SuperScript VILO cDNA Synthesis Kit (Cat # 11,754,050). The cDNAs were diluted 20 times and reverse transcription-quantitative (RT-qPCR) was performed using SYBR Green Real-Time qPCR Master Mix (Thermo Fisher Scientific) in a 10 μl reaction volume. The primer sequences used for RT-qPCR reactions are listed in Table S2. Relative mRNA expression was calculated by using 2^−ΔΔCt^ method and normalized to that of 18S rRNA.

### Migration and invasion analysis

The migratory ability of U87 cells following different treatments was evaluated using 24-well Transwell plates (Corning Costar) with 8 μm pore membranes. For each sample, 1 × 10^5^ cells were seeded on the upper part of transwell and 250 ng/ml of epidermal growth factor supplemented complete media (DMEM/F12 + 10% FBS) was used as chemoattractant in the lower part of chamber and cells were incubated for overnight under tissue culture conditions. Following incubation, upper side of Transwell membranes was wiped with a cotton swab to remove nonmigratory cells and fixed by using 4% paraformaldehyde. Membranes were stained using 1% crystal violet stain for 10 min followed by washing and microscopy analysis. Quantification of migratory response was performed after solubilizing the crystal violet dye in 10% acetic acid and measuring absorbance at 595 nm.

For invasion analysis, identical protocol as described above was used except the transwell membranes were coated with Matrigel (1:50 dilution) for 1 h at room temperature before seeding cells.

### Chromatin immunoprecipitation

Cells were cross-linked with 1% formaldehyde, lysed, and sonicated using diagenode bioruptor for 10 min at 30 s on/off cycles for shearing of genomic DNA with an average size of ∼500 bp. The samples were diluted in ChIP dilution buffer and immunoprecipitated using antibodies as provided in the list. Immunoprecipitation using normal immunoglobulin G (IgG) (Cat. no. PP64B; Millipore) was used as the negative control for all experiments. The protein/DNA complexes were collected either using protein A or protein G Magna ChIP magnetic beads (Millipore), reverse-crosslinked and DNA purified using Qiagen PCR purification cartridges. Purified DNA was analyzed by RT-qPCR using SYBR Green Real-Time PCR Master Mix (Thermo Fisher Scientific) in a 10 μl reaction volume. The sequences of the primers are provided in Table S3. The information on antibodies is provided in the list of antibodies in Table S1.

### Apoptosis analysis

TUNNEL assay for apoptosis was performed using TACS 2 TdT-DAB *In Situ* Apoptosis Detection Kit (Trevigen) following protocol provided with the kit and cells imaged using EVOS XL-Core phase-contrast microscope at 20× magnification.

### Co-immunoprecipitation

Cells were washed twice with 1× PBS and lysed using freshly prepared Co-IP lysis buffer supplemented with 1× Halt protease and phosphatase inhibitor cocktail (Cat. no. 1861284; Thermo Fisher Scientific), incubated for 30 min at 4 °C on a rotating platform. After incubation, lysates were centrifuged at 12,000 rpm in a 5417R Eppendorf centrifuge for 15 min at 4 °C. The supernatant was transferred to a clean tube and placed on ice. Protein concentration was quantified and measured using the bicinchoninic kit (BCA protein assay kit, Cat. no. 23225; Thermo Fisher Scientific). For each immunoprecipitation reaction, 1 mg of protein lysate was mixed with 5 μg of either immunoglobulin G antibody (negative control) or experimental antibody in a total volume of 400 μl and incubated overnight at 4 °C on a rotating platform. Post overnight incubation, 40 μl of precleared Protein A agarose beads (Cat. no. 16–125; Millipore) were added and incubated for at least 2 h at 4 °C on a rotating platform. After incubation, the bead-antibody-protein lysate complex was washed four times with Co-IP buffer. In each wash, beads were mixed gently on a rotating platform for 3 min, the antibody-bead-lysate complex spun down using micro spin, and the supernatant was carefully discarded without disturbing the agarose beads. Beads were resuspended in 40 μl of 2× loading dye sample buffer and denatured at 95 °C for 5 min and spun down after elution to separate protein complex from agarose bead. After spin, the protein complex in the supernatant was transferred to a clean tube and stored at −80 °C for further use for Western blotting. The blots were quantitated using ImageJ software. The information on antibodies is provided in the list of antibodies in Table S1.

## Data availability

Raw and processed RNA-seq data were deposited to the NCBI GEO database under accession number GSE234473.

## Supporting information

This article contains supporting information.

## Conflict of interest

The authors declare that they have no conflicts of interest with the contents of this article.
