# Supplementary material for: O-GlcNAc transferase promotes glioblastoma by modulating genes responsible for cell survival, invasion, and inflammation
Source: J Biol Chem. 2023 Sep 9;299(10):105235. doi: 10.1016/j.jbc.2023.105235 (PMC10570119; doi:10.1016/j.jbc.2023.105235)
Supplement: Supporting Figures S1–S3 and Tables S1–S3 [file mmc1.pdf]

Figure S1. Identification of differentially expressed genes due to OGT and OGA knockdown in U87 cells. Number of genes up/down-regulated in sh-OGT and sh-OGA U87 cells compared to vector control (Ctrl) with p-values of ≤0.05 and a fold change of two or more.

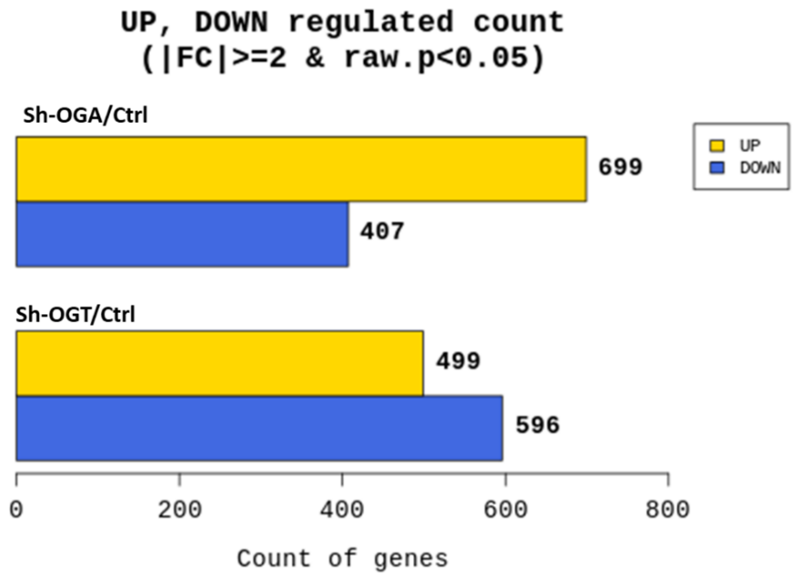

Figure S2. Transcription factor (TF) enrichment analysis identifies BRD4 enriched at genes downregulated due to OGT knockdown in U87 cells. A web based transcription factor enrichment analysis (TFEA) tool (https://www.iib.uam.es/TFEA.ChIP/) was used to identify TF enrichment on the list of genes significantly downregulated (A) and upregulated (B) (≥ 2 folds with p≤ 0.5) due to OGT depletion.

A

TR	Wilcoxo...	Wilcoxon...	Z-score	max AUC	Relative...	Irwin-Hall ...
BRD4	10.852	9.807e-28	0.945	0.891	0.028	**1.031e-04**
TP53	2.914	1.783e-03	1.274	0.888	0.028	**1.031e-04**
MED1	5.818	2.973e-09	1.011	0.878	0.029	**1.070e-04**
CHD4	3.104	9.531e-04	1.751	0.847	0.034	**1.792e-04**
FOSL2	7.428	5.493e-14	1.542	0.831	0.036	**2.154e-04**
BCL3	7.903	1.366e-15	1.054	0.850	0.039	**2.633e-04**
FOSL1	2.820	2.403e-03	1.804	0.833	0.045	**4.083e-04**
YAP1	1.743	4.069e-02	1.295	0.896	0.046	**4.282e-04**
ELK4	3.271	5.352e-04	0.828	0.872	0.052	**6.506e-04**
NFE2L2	2.887	1.944e-03	2.015	0.821	0.053	**6.640e-04**
JUN	4.259	1.025e-05	1.638	0.816	0.053	**6.777e-04**
PMEL	2.221	1.318e-02	1.742	0.837	0.053	**6.777e-04**
MYC	8.280	6.183e-17	0.643	0.885	0.059	**9.053e-04**

B

TR	Wilcoxon T...	Wilcoxon P...	Z-score	max AUC	Relative rank	Irwin-Hall P...
REST	7.640	1.084e-14	2.947	0.620	0.014	**1.147e-05**
TEAD4	4.427	4.770e-06	2.852	0.605	0.021	**4.079e-05**
TRIM28	4.244	1.098e-05	3.698	0.581	0.025	**6.867e-05**
RAD21	10.239	6.642e-25	2.305	0.631	0.032	**1.425e-04**
POLR3D	4.361	6.468e-06	2.706	0.577	0.033	**1.681e-04**
CTCF	29.568	1.953e-192	2.240	0.618	0.038	**2.561e-04**
ZNF649	1.707	4.393e-02	4.450	0.627	0.040	**2.782e-04**
SMC3	7.853	2.027e-15	2.191	0.629	0.041	**3.097e-04**
SMC1A	7.439	5.080e-14	2.212	0.625	0.041	**3.097e-04**
ZFP57	2.405	8.078e-03	2.516	0.620	0.041	**3.179e-04**
TEAD1	3.524	2.129e-04	2.476	0.583	0.043	**3.703e-04**
NR3C1	8.661	2.344e-18	2.282	0.584	0.044	**3.890e-04**

Figure S3. OGT and OGA inhibition do not affect BRD4 O-GlcNAcylation. (A) Co-immunoprecipitation (Co-IP) was performed on cell lysates from DMSO (Ctrl) and OGT inhibitor, ST078925 (left blot) and DMSO (Ctrl) and OGA inhibitor, TMG (right blot) treated cells using anti-BRD4 antibody followed by western blotting of IP'd samples using anti-O-GlcNAc antibody. IP with IgG was used as control. Input is the total cell lysate. (B) Densitometric quantitation of western blots from panel A. (C) Western blot analysis of BRD4 in Ctrl (DMSO treated) and OGT inhibitors, (ST078925 and OSMI-1 treated) U87 cells. (D) Densitometric quantitation of western blots from panel C. GAPDH was used as loading control. Data represent mean of three biological replicates ± SD. Asterisks represent differences being significant (**$p < 0.01$). ns (not significant).

A

B

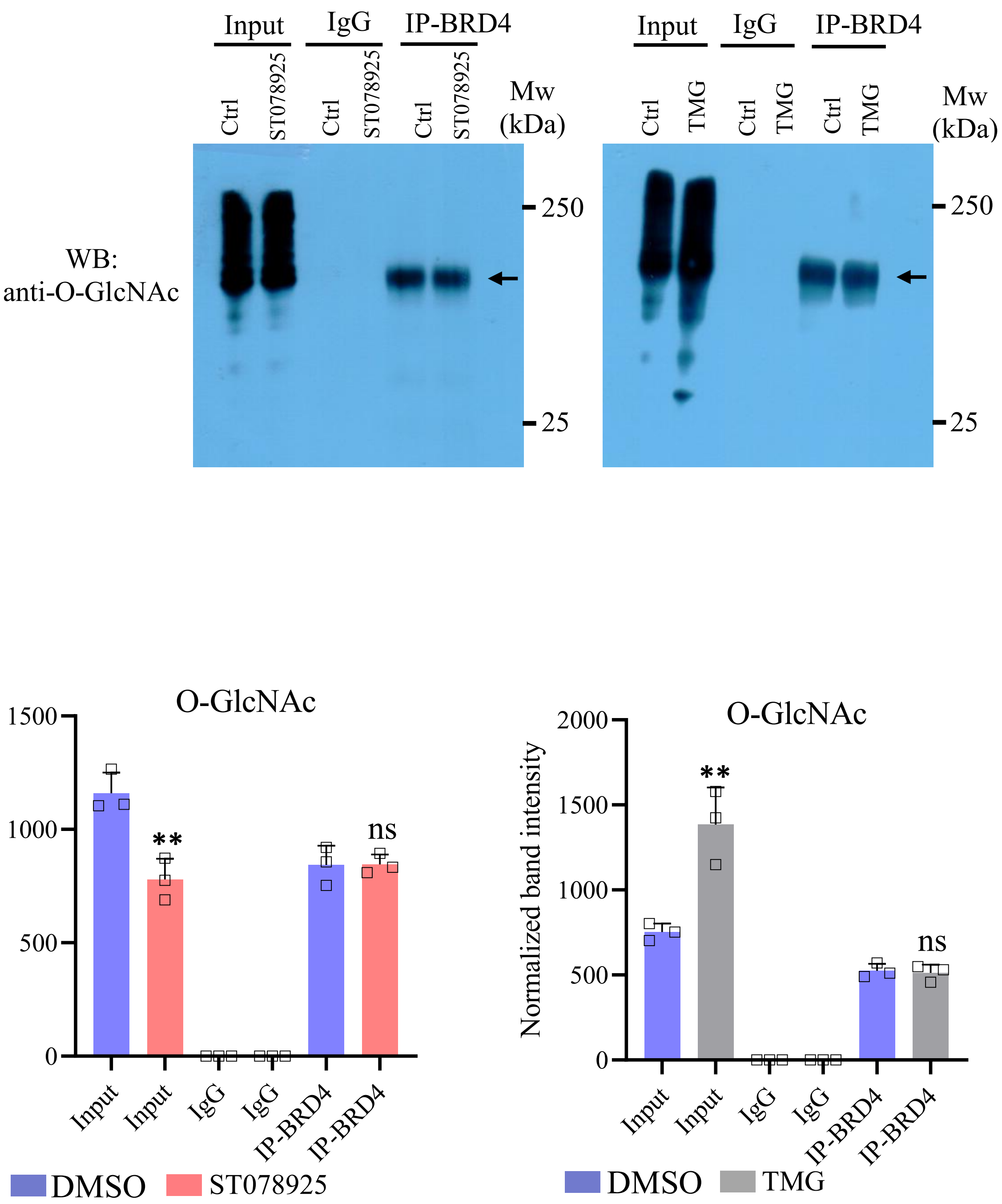
Input
IgG
IP-BRD4
Ctrl
ST078925
Ctrl
ST078925
Ctrl
ST078925
Mw (kDa)
250
25
WB: anti-O-GlcNAc
Input
IgG
IP-BRD4
Ctrl
TMG
Ctrl
TMG
Ctrl
TMG
Mw (kDa)
250
25
O-GlcNAc
Normalized band intensity
1500
1000
500
0
**
ns
Input
Input
IgG
IgG
IP-BRD4
IP-BRD4
DMSO
ST078925
O-GlcNAc
Normalized band intensity
2000
1500
1000
500
0
**
ns
Input
Input
IgG
IgG
IP-BRD4
IP-BRD4
DMSO
TMG

C

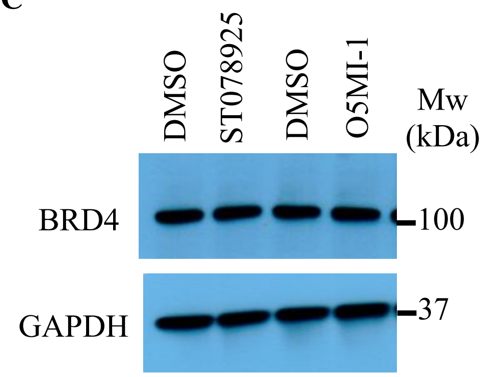

D

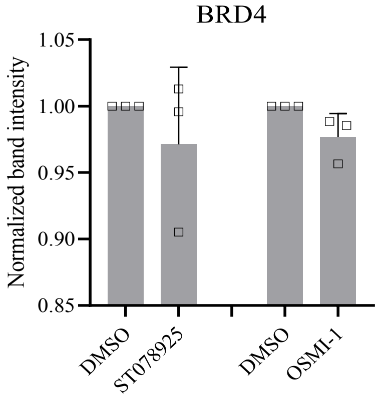

Supplementary table 1: A list of antibodies used in this study.

**Antibody**	**Source**	**Catalog No.**
OGT Polyclonal antibody	Proteintech	11576-2-AP
MGEA5 Polyclonal antibody	Proteintech	14711-1-AP
BrdU Rabbit pAb	Abclonal	A20304
Anti-RNA polymerase II subunit B1 (phospho CTD Ser-2) Antibody, clone 3E10	Sigma, Millipore	04-1571
CDK9 (C12F7) Rabbit mAb	Cell Signaling Technology	2316S
TRAP220 Antibody (H-7)	Santa Cruz	sc-74475
TBP (D5G7Y) Rabbit mAb	Cell Signaling Technology	12578
GAPDH (14C10) Rabbit mAb	Cell Signaling Technology	2118
Anti O-Linked N-Acetylglucosamine antibody [RL2]	Abcam	ab2739
Ubiquityl-Histone H2B (Lys120) (D11) Monoclonal Antibody	Cell Signaling Technology	5546

Histone H3K27ac (Acetyl H3K27) Polyclonal antibody	Epigentek	A-4708
Go-ChIP-Grade™ Purified anti-RNA Polymerase II RPB1 Antibody (8WG16)	BioLegend	664912
Anti-RNA polymerase II subunit B1(phosphor CTD Ser-5) clone 3E8	Millipore	04-1572
Purified polyclonal Rabbit IgG	BioRad	PRABP01
Peroxidase AffiniPure Goat Anti-Mouse IgG (H+L)	Jackson Immuno Research	115-035-166
Peroxidase AffiniPure Goat Anti-Rabbit IgG (H+L)	Jackson Immuno Research	111-035-144
BRD4 (E2A7X) Rabbit mAb	Cell Signaling Technology	13440
Rabbit anti-ELL2 Antibody	Bethyl Laboratories	A302-505A
Akt (pan) (C67E7) Rabbit mAb	Cell Signaling Technology	4691
Phospho-Akt (Thr308) Antibody	Cell Signaling Technology	9275
Phospho-Akt (Ser473) Antibody	Cell Signaling Technology	9271
Stat3 Antibody (F-2)	Santa Cruz	sc-8019
p-Stat3 Antibody (B-7)	Santa Cruz	sc-8059
HEXIM1 (D5Y5K) Rabbit mAb	Cell Signaling Technology	12604
Pan Phospho-Serine/Threonine Rabbit pAb	Abclonal	AP0893

Supplementary table 2. A list of oligonucleotide sequences used for RT-qPCR.

Primer Name	Sequence (5' to 3')
GAP43-F	ACCATGCTGTGCTGTATGAG
GAP43-R	GTGTTATGACCTCGTCACCCA
IL1B-F	CAGAAGTACCTGAGCTCGCC
IL1B-R	AGATTCGTAGCTGGATGCCG
MMP1-F	TGTGGTGTCTCACAGCTTCC
MMP1-R	CGCTTTTCAACTTGCCTCCC
CXCL1-F	TCACAGTGTGTGGTCAACAT
CXCL1-R	AGCCCCTTTGTTCTAAGCCA
RAP1B-F	GGTGACAGCGTGAGAGGTTC
RAP1B-R	GACTTTCCAACGCCTCCTGA
TGM2-F	ATTCCCTCTCCTGCCCAGAT
TGM2-R	AGGGAGCTGGATTCCCTGAT
NAT2-F	GTTGCTGGCCAAAGGGATCAT
NAT2-R	AGCCTCTAAGCCCAACTCCAT
SERPINE1-F	AGAGCGCTGTCAAGAAGACC
SERPINE1-R	AGTTCTCAGAGGTGCCTTGC
SERPINB2-F	TCTCAGAGGAGCATTGCCCG
SERPINB2-R	AGTGCAAGAAATGCTGGTTG
PCNA-F	GCCCTGGTTCTGGAGGTAAC
PCNA-R	TAGCTGGTTTCGGCTTCAGG
MET-F	CGACAGCTGACTTGCTGAGA
MET-R	AGGTATCTCGGTGCCCAGTC
GFPT2-F	GACAATTGAGCTGCCCGATC
GFPT2-R	TGGTGATGGTCTTGTCACGG
ELL2-F	ATGTGAAGCTCACCGAGACG
ELL2-R	CTGCCCTGAGGGTTGTCTTT
AXL-F	CACCCCAGAGGTGCTAATGG
AXL-R	GAAGGTTCCTTCACTGGGCG

Supplementary table 3. A list of oligonucleotide sequences used for ChIP-qPCR.

Primer Name	Sequence (5' to 3')
HGF-F	AGTTTGGTCACCCACATGGT
HGF-R	AGCCGACTGGCTCTTTTAGG
MET-F	GAGACCTGACTGCTGTTCCA
MET-R	TTCTAGTTTCGTCCTCGCCG
PCNA-F	TACCCACTTCCAAGCCATGT
PCNA-R	GCCGTCGCTAGTACTTGTT
SERPINE1-F	AGTGACCTGGTTCGCCAAAG
SERPINE1-R	ACCCAAAAGCCTAGGACCC
SERPINB2-F	GGGAGGGGCAAAGCTGTATAA
SERPINB2-R	TCTCTGAGTTGCTGTCTGACG
ELL2-F	CTTGCTTGTCCACACCCCTT
ELL2-R	GTAGAAACAGTGGCATGCGG
GAP43-F	AACCCGAGTACAGTATTTCC
GAP43-R	AGCTGGGATGCAACCATCA
HAS2-F	ATGGGGCTTCTACACGTTCC
HAS2-R	AAACGTCCGCGCTGAATACT
TGM2-F	TGATACTCACCCTCGGCCAT
TGM2-R	ATAACTAGCGCCGCTCTCC
ELK3-F	TGTCAGCATGGAAAGTCGGG
ELK3-R	ACAGGCAGAAGCCCTGTTAC
NAMPT-F	TCTGCCGCAGGATTCATCTC
NAMPT-R	CCGACCGAGCAGTGACTTA
GFPT2-F	GGGGCTTAGGGCAACCAAC
GFPT2-R	GTTTCACAGACGGGAAAGCG
MMP1-F	CAGTGCAAGGTAAGTGATGGC
MMP1-R	TCTGGAAGGGCAAGGACTCTA
IL1B-F	CCCTCGCTGTTTTTATGGCT
IL1B-R	TCCCCTAAGAAGCTTCCACC
NAT2-F	GCGGGGCGATTTGCATTTCT
NAT2-R	AGCCCAAAAGCCATCCCTGA
